# A real-time video-based social network platform for online ultrasound education

**DOI:** 10.1186/s12909-022-03220-1

**Published:** 2022-03-08

**Authors:** Yi Zhang, Shenyi Li, Xiangdang Long, Xi Li

**Affiliations:** Department of Ultrasound Medicine, Hunan Provincial People’s Hospital, First Affiliated Hospital of Hunan Normal University, No. 61, Jiefang West Road, Furong District, Changsha City, Hunan Province China

**Keywords:** Live online, Ultrasound medicine, YiQixiu, Questionnaire, Education

## Abstract

**Background:**

In the post-pandemic era, traditional methods of professional development for ultrasound practitioners are insufficient, and it is therefore imperative to explore a new avenue for continuing education. This article explores the role of the real-time video-based social networks for medicine combined with e-enterprise to train ultrasound practitioners.

**Methods:**

We created a live broadcasting room on the real-time video-based social networks for medicine and imparted online education on ultrasound usage with “YiQixiu” as the transmission carrier. We developed a satisfaction questionnaire for the online class in real time, and tested the validity and reliability of the questionnaire. Descriptive statistical analysis was used (*P* ≤ 0.05 indicates significance).

**Results:**

The landing page on YiQixiu was mainly concentrated in the Hunan Province, accounting for 56% of visitors. The total number of people watching online real-time lectures was 32,344; the maximum number of fixed attendance was 17,000, and the minimum number was 3,000. The questionnaire met the needs of this study, with a reliability value of 0.93. The participants were from 18 provinces, 4 autonomous regions, and 4 municipalities directly under the central government.

**Conclusion:**

The real-time video-based social networks for medicine combined with the YiQixiu live platform is a good method for imparting ultrasound medical education online.

## Background

Ultrasound as a diagnostic facility is available at all large and small hospitals, imaging centers, community health centers, multispecialty clinics, and private practice offices, but the diagnostic yield is highly uneven. Added to this is the challenge of obtaining expert opinion and senior input in difficult, rare or complicated cases. New developments and current technology are often not available, leaving patients at a disadvantage. While lifelong learning, continuing medical education, training courses, and conferences have been common modes of professional development, these are often not sufficient to meet the needs of most ultrasound practitioners, especially those working at a grassroots level or in remote areas. In some cases, continuous training is not possible owing to staff shortages, hierarchal systems, or financial constraints. In natural disasters or certain ‘force majeur’ situations, such as the COVID (Corona Virus Disease) pandemic, the stark disadvantages of ultrasound diagnosis and knowledge not being disseminated optimally are even more prominent. With the challenges posed by the COVID-19 pandemic there also came ample opportunities to utilize technology and the Internet to take education to the masses, and in China this emerged as the "Internet plus Education" plan, with a promising arena for research [1]. With the near abolition of offline or physical conferences and continuous professional development programs, a purely online live stream model, and later a hybrid model, became the need of the hour. This transition was enabled by the technological progress of the Internet, which made it not only feasible but also comfortable and therefore an acceptable mode of teaching–learning [2]. The ability to remain interactive, as well as being cost effective, has led to its mass appeal [3]. Not only this, but even in higher education the avenues for continuous professional development have increased manifold [4]. In order to move towards optimization, it is imperative for individuals to receive training (knowledge) in ultrasound diagnosis in a new medium. Educators worldwide seized the tremendous potential of online platforms to reach out to and empower medical students in their pursuit of knowledge [5]. Video based platforms enhance the teaching–learning process by simulating the real world experience as closely as possible. Furthermore, medical students should also be empowered in digital formats like telehealth, including patient-physician remote interaction [5]. Several researchers have also taken it a step forward by using these platforms on social media networks for wider dissemination and value [6]. This article explores the role of the real-time video-based social networks for medicine in conjunction with the new model of YiQixiu for live online lectures in ultrasound medicine so that professional expertise may be disseminated more efficiently in real time with the help of modern technology.

## Methods

**Fig.1:**
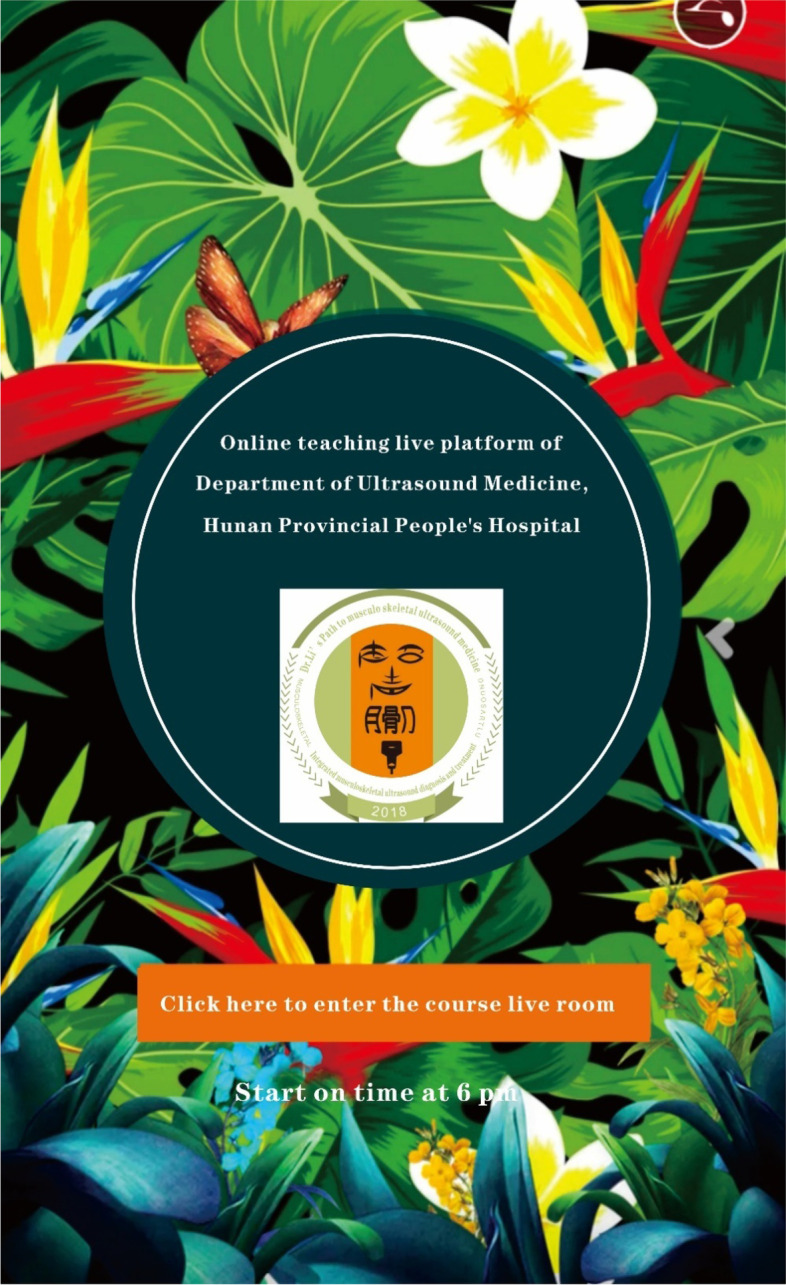
YiQixiu notification home screen screenshot

An individual real-time video-based social networks for medicine account was created, the nickname modified, and log in provided in the prescribed format. The administrator for the real-time video-based social network for medicine was assigned, a live room name constructed, and a room ID number generated. Simultaneously, a login to the official website of YiQixiu was facilitated with the YiQixiu notice (see Fig. 1), and the real-time video-based social networks for medicine link was inserted on the YiQixiu page; the course schedule and instructor panel were shared in advance every Wednesday on another page. The real-time video-based social networks for medicine live room (including the live room name and ID number) was promoted by the platform administrator through various channels on the network (such as WeChat, QQ ultrasound practitioners’ group, Weibo, Douyin, etc.), and the YiQixiu notice was forwarded with detailed instructions about entering the lecture room. Physicians who needed to study ultrasound medicine could open the YiQixiu notice at a prescribed time and then directly click the live room link on the page for real-time lectures.

The teaching materials were sourced from the department in the form of short lectures on related topics, including ultrasound diagnostics, new technologies, continuous professional development, discussion of difficult cases, and teaching rounds. The lecturers and resource persons comprised mainly the department faculty, invited experts in ultrasound medicine at both national and international levels, and other clinical experts in the same hospital.

Domestic ultrasound practitioners and other clinicians, international experts, and related non-professionals were included as trainers. On-site lecturers belonged to the Department of Ultrasound Medicine and other clinical departments of the same hospital. Graduate students, interns, and nearby attendees were present on-site.

The YiQixiu system automatically records and counts the relevant data. We extracted the total page visits, unique visitors, re-shares, visit regions, and access device distribution from April 26, 2018 to March 14, 2020 from the analysis data column of YiQixiu. YiQixiu also has a message function, which can record the satisfaction scores and feedback from both trainers and trainees. We used the real-time video-based social networks for medicine to obtain a fixed number of clients, and the number of real-time lectures and lectures per live online broadcast.

We formulated a live online class satisfaction questionnaire (see Table 1) on the basis of carefully researched and relevant documents, and we used this raw client data to publish the survey questionnaire; this method was useful towards understanding, analyzing, and investigating the ideas of the participants.

**Table 1:** Do you like the dissemination of ultrasound medical knowledge in the form of online live broadcast? (*n* = 181)

Options	Number of people	Percentage%
Like	178	98.34%
General	1	0.55%
Uncertain	0	0%
Dislike	2	1.11%

Descriptive statistical data analysis was used (*P* ≤ 0.05 indicates significance). Validity and reliability were tested based on expert evaluation, correlation analysis, and correlation coefficient to obtain the reliability score of the questionnaire.

This study was approved by the Medical Ethics Committee of Hunan People’s Hospital (First Affiliated Hospital of Hunan Normal University) (No. 2020–49), all methods were carried out in accordance with relevant guidelines and regulations. The written informed consent was obtained from all the participants for involvement in the study.

## Results

From April 26, 2018 to March 14, 2020, the total number of visits to the YiQixiu notification page reached 52,886, with a daily maximum of 1,053, a minimum of 1, and an average of 86. The number of independent visitors reached 30,216; the highest daily number of visits was 596, the lowest 1, and the average 51. The regions visited were mainly concentrated in Hunan Province, Henan, Jiangsu, Hebei, Shandong, Guangdong, Jiangxi, Sichuan, Beijing, and Zhejiang. Hunan Province provided the largest proportion, accounting for 56%. The preferred channel was mainly WeChat, accounting for 100%. The access device data showed that the major views were on Android (74%) and Apple (24%), and the main models were Huawei (35%), Apple (25%), OPPO (9%), Vivo (9%), Xiaomi (7%), and Samsung (2%). The total number of re-shares of YiQixiu was 354. There were 1,020 messages in the message column of YiQixiu, and the audience satisfaction was 98%. The main comments were that the listeners felt that the live broadcast schedule was not reasonable, some live broadcasts were not smooth, and the sound quality was poor. There were also suggestions that the lecturer should begin with basics and standardize the professional videos. During and post COVID, efforts have been made to adjust the live broadcast time and to continually improve the quality and standardize the professional content.

An online live class satisfaction questionnaire was created after meticulous planning. A total of 195 questionnaires were distributed online, with 181 validated and 14 non-validated questionnaires. The participants in the survey were from 18 provinces, 4 autonomous regions, and 4 municipalities in China.

Results of the survey form are provided below.

Table 1 shows that almost all participants in the survey liked the dissemination of ultrasound knowledge through the online live broadcast. A small number of participants who disliked the online format likely prefer the traditional classroom. Online education could potentially be used for ultrasound training going forward.

Table 2 shows that almost all participants were satisfied with the course content and design, and 69.61% were very satisfied. The course content ranged from basic theories to actual cases, to new advances and new technologies, and from easy to difficult. The lectures were classified and explained according to the subject design.

**Table 2:** Are you satisfied with the content design of the online live broadcast of the platform? (*n* = 181)

Options	Number of people	Percentage%
Very satisfied	126	69.61%
Satisfied	52	28.73%
General	3	1.66%
Very dissatisfied	0	0%

Table 3 shows that the participants felt that the online live teaching mode was helpful for improving the diagnosis and treatment capabilities of ultrasound practitioners, especially for those in remote areas and those who did not have the time and opportunity to participate in advanced studies.

**Table 3:** Does the online live teaching model help improve the diagnosis and treatment capabilities of ultrasound practitioners? (*n* = 181)

Options	Number of people	Percentage%
Very helpful	157	86.74%
Somewhat helpful	23	12.71%
General	1	0.55%
Did not help, and played a negative role	0	0%

Table 4 shows that problems with interference were uncommon, and attention/concentration was maintained except in cases of poor network signals and webpage pop-up interference.

**Table 4:** Is the online live class hampered by other networks, and does it impair concentration? (*n* = 181)

Options	Number of people	Percentage%
Very often	12	6.63%
Often	90	49.72%
Sometimes	2	1.11%
Almost never	77	42.54%

Table 5 shows that the majority of participants believed that watching online live classes could stimulate the desire to gain professional knowledge.

**Table 5:** Do you feel that live online classes can stimulate your desire to gain professional knowledge? (*n* = 181)

Options	Number of people	Percentage%
Agree	162	89.50%
General	9	4.97%
Uncertain	10	5.53%
Disagree	0	0%

Table 6 shows that all the participants in the questionnaire supported the continued use of the online live broadcast platform for the dissemination of ultrasound knowledge and expertise, with the wide majority highly supportive.

**Table 6:** Do you support us in continuing the use of the online live broadcast platform to spread ultrasound knowledge and expertise? (*n* = 181)

Options	Number of people	Percentage%
Very supportive	163	90.06%
Supportive	15	8.30%
Uncertain	3	1.66%
Not supportive	0	0%

Table 7 shows that all the participants in the questionnaire were willing to recommend our online live courses to professionals who were in need of training resources. (‘Not willing’ was taken to imply that active promotion by users was not agreed to, whereas ‘not support’ would be that the method itself was unacceptable.)

**Table 7:** Are you willing to recommend our live online classes to professionals in need? (*n* = 181)

Options	Number of people	Percentage%
Willing	174	96.13%
Not willing	3	1.66%
Uncertain	4	2.21%
Not support	0	0%

Table 8 shows that most participants were driven by the ultrasound professional team to choose to watch our online live courses, accounting for 67.40%, followed by industry reputation and trial class.

**Table 8:** What factors prompted you to choose to watch our live online class? (*n* = 181)

Options	Number of people	Percentage%
Ultrasound professional team	122	67.40%
Industry reputation	20	11.05%
Satisfied with the trial class	21	11.60%
Recommended by other	18	9.95%
Ultrasound professionals		

## Discussion

Ultrasound medicine as an independent and complete system of diagnostics is not only highly theoretical, but also technical. Ultrasound practitioners must be ability-oriented, with comprehensive high-quality training. Not only must a new mode of knowledge transfer be integrated into the system, but capacity building should also be enhanced, especially for grassroots workers and those in remote areas. This is even more important in the post COVID era, where real-time, prompt, fast, and effective dissemination of professional knowledge and skills is required. Thus, exploring new modes of online live broadcast is urgent.

With the growth of Internet technologies, especially big data and cloud computing, and the popularization of smart terminals, it is possible to transfer expertise online. The new generation of media real-time video-based social networks for medicine was born in 2008. It is called “Waiyin” [7] on the Internet and is the abbreviation of “yuyin” in pidgin Chinese. At present, with its unique form of presentation, the real-time video-based social networks for medicine provides voice and video services in online real-time interactive classrooms, and learners can break time and space constraints to learn and interact. The popularity of the Internet has promoted the development of novel network communication technologies. Online live broadcasting is one of the largest ways to disseminate information. It is an interactive and quasi entertainment form based on real time video streaming that supports dialogue between anchors and users. It mainly relies on webpage or client technology to build a virtual live online room, with a platform facilitating real-time performance for the anchor [8]. Online live broadcasting, as a popular voice tool for online instant communication, has changed traditional online education methods and approaches, conforming to the current trend of the Internet, and has the advantages of a strong interface, rich teaching–learning channels, and high-definition video points. Based on this, our department successfully created a real-time video-based social networks for medicine room and generated a room ID number in 2017. The live broadcast room was renamed, using YiQixiu as the live broadcast platform communication carrier. This was done after logging in to the official website of YiQixiu, creating the Yiqixiu notice, and inserting the real-time video-based social networks for medicine link on the page. On the subsequent pages, the teaching schedule and training panel were displayed, along with the subject, teacher, and host information. The platform administrator or department secretary shared the YiQixiu notification link or WeChat QR code through various channels on the network (WeChat, QQ, Weibo, Douyin, etc.), with detailed instructions to enter the live room and avail of the lesson. Students who needed to obtain expertise in ultrasound medicine could click on the link to the online live lesson after receiving the YiQixiu notification link, collect it on WeChat, open the YiQixiu notification link or WeChat at the prescribed time, and then click the link to listen to the lesson in real time. The live teaching materials were sourced from the department in the form of short lectures incorporating basic knowledge of ultrasound medicine, professional development, new skills, discussion of difficult cases, and teaching rounds. The trainers were mainly from the department (Department of Ultrasound Medicine) and related specialties in the same hospital. Some ultrasound experts were also invited from inside and outside the province. This entire program was reported for the first time on the official website of the Hunan Provincial Health Commission in the year of its inception (2017). In China, this new model of an online live broadcast platform combined with YiQixiu to disseminate and impart ultrasound training is still considered unique. In a little more than two years of continuous use of this medium, a large number of interested trainees have been accommodated. Expert senior input has further enhanced the value of this program.

YiQixiu has the following advantages: extremely simple operation, rich expression, abundant applications, and the ability to automatically store and disseminate analysis data in real time. All kinds of complex and mundane knowledge points that can be produced and displayed on the computer devices are transferred to more convenient and portable mobile phones. Learners may choose their preferred device according to their needs without time or space limitations. Beginning in 2018, this platform was promoted in our setup as a novel educational medium, and except for holidays this program continues to be live streamed online every Wednesday at 6 pm local time. The YiQixiu notification link is shared one day before the lecture by the multiparty network platform. All online live courses are free to watch. At present, our real-time video-based social network for medicine is fixed at 1,590 fans/clients/attendees (live room ID number 1963199794). The number of real-time live lecturers has reached 60 and the minimum is 25, including members of the Department of Ultrasound Medicine and some from other clinical departments. Graduate students, independent trainees, interns, and learners from nearby foreign hospitals usually join on-site.

From the results of our questionnaire, most questionnaire participants learned about our online live broadcast program through the ultrasound professional team, and they provided very encouraging feedback for its continued dissemination. They unanimously expressed the view that the online live broadcast classes were robust in design and content and could stimulate their desire to acquire professional knowledge and skills. The participants were also willing to promote our online live broadcast platform to other professionals. Some respondents felt that the online courses were susceptible to interference from other networks and affected their concentration (57.46%). A domestic research survey shows that when using the WeChat platform to implement the flipped classroom process, 70% of the students are easily distracted by other network notifications [9]. The main points of contention were poor network signals and advertising pop-ups. In the Internet era, and especially post-pandemic, live online courses have become a more popular (and oftentimes the only) way of disseminating knowledge, and are eminently worth exploring further.

Keeping the importance and urgency of the current situation in mind, online live broadcast teaching platforms can promote expertise in ultrasound practice among medical professionals nationwide (especially in the province of Hunan). With the emergence and popularization of newer and better streaming technologies, and the philosophy of lifelong learning, disparities in training and practice of ultrasound medicine can be overcome. Although this learning platform is a break from tradition, it meets the needs of ultrasound practitioners- regardless of time and place- with diversified and customizable learning, reduces their learning costs, and makes learning more autonomous. Disruption, adaptation, and growth are buzzwords in this new learning era and almost all spheres of education and commerce are following this trajectory. From a global perspective, Madrigal et al. [6] used Facebook and YouTube to create a platform called pathCast, which broadcasts live pathology lectures to international medical professionals online, with good results. Therefore, live online broadcasting has become an invaluable method in education and the dissemination of new medical technologies [10]. Thus, adoption of new technology could benefit both patients and practitioners in (ultrasound) medicine.

## Conclusion

The real-time video-based social networks for medicine combined with the YiQixiu live platform is a good method for teaching ultrasound medicine online.

## Data Availability

The raw data supporting the conclusions of this article will be made available by the corresponding author, without undue reservation.

## Abbreviation

COVID: Corona Virus Disease
